# Radiation damage in sub-Ångström resolution macromolecular crystallography: a low-dose study

**DOI:** 10.1107/S205979832600269X

**Published:** 2026-04-13

**Authors:** Gleb Bourenkov, Elham Paknia, Claus Flensburg, Rasmus Fogh, Peter Keller, Clemens Vonrhein, Gérard Bricogne, Ashwin Chari

**Affiliations:** a European Molecular Biology Laboratory Hamburg Unit, Notkestrasse 85 22607Hamburg Germany; b Department of Structural Dynamics Max Planck Institute for Multidisciplinary Sciences Am Fassberg 11 37077Göttingen Germany; c Global Phasing Limited 9 Journey Campus, Castle Park CambridgeCB3 0AX United Kingdom; d Research Group for Structural Biochemistry and Mechanisms Max Planck Institute for Multidisciplinary Sciences Am Fassberg 11 37077Göttingen Germany; Institut de Biologie Structurale, France

**Keywords:** radiation damage, sub-Ångström resolution, Fe–S_4_ cluster bond lengths, aspherical electron densities

## Abstract

Sub-Ångström macromolecular crystallography allows the construction of structural models without any prior chemical knowledge and stereochemical restraints, making it possible to visualize deviations from peptide planarity, distortions of the planarity of aromatic ring systems and subtle alternate conformation networks. It also holds the promise of observing aspherical density features and other quantum-mechanical phenomena. However, the degree to which radiation damage might affect the details that can be discerned from biological structures at such resolutions has remained unknown. To address this, we report here the first study of radiation-damage effects at sub-Ångström resolution on *Pyrococcus abyssi* rubredoxin. Our data, collected at 100 K, indicate that the oxidized (Fe^3+^) state of the Fe atom is preserved to a large degree at a dose of 50 kGy, whereas at 1 MGy it is partially reduced to the Fe^2+^ state. Isomorphous difference maps reveal extensive conformational changes at 1 MGy that are most likely coupled to the reduction of Fe^3+^. At 1 MGy, hydrogen densities that are visible at 50 kGy are preserved, but are distinctly blurred by dose. These findings suggest that the ‘global’ radiation damage in reciprocal space and the associated blurring of electron density in real space proceed through small structural changes that are much more extended than the local damage at specific sites which is normally seen at lower resolution. Thus, the ‘global’ and ‘specific’ damage could be viewed as two sides of the same coin. Our study highlights the unique benefits of using low-dose data-collection protocols on large crystals fully bathed in a large ‘top-hat’ beam with a uniform fluence profile for accurate sub-Ångström structural investigations on macromolecules, as only this experimental configuration can deliver the uniform spatial distribution of dose necessary to resolve the fine details of progressive radiation damage: a capability for which we propose the term ‘resolution in dose’.

## Abstract 2

This first systematic radiation-damage study at 0.55 Å resolution reveals extensive small conformational changes in response to dose. The findings demonstrate the benefits of using large crystals fully bathed in a large ‘top-hat’ beam with a uniform fluence profile, together with low-dose data-collection protocols, for sub-Ångström structure investigations on macromolecules.

## 1. Introduction

Radiation damage is of paramount concern in modern structural studies of biological macromolecules. Radiation damage occurs through the absorption of ionizing radiation (X-rays and electrons; Garman & Weik, 2023 ▸). In macromolecular crystallography, the widespread use of intense synchrotron sources has forced researchers to mitigate radiation-damage effects often on a greater scale than previously required. This affects both data quality in X-ray crystallography and crucial practical aspects, such as sample lifetime and the maximum resolution attainable in a diffraction experiment (Sliz *et al.*, 2003 ▸; Ravelli & Garman, 2006 ▸). Critically, the accumulated dose (measured in Gy = J kg^−1^) not only degrades diffraction quality, but potentially alters the very nature of the sample and therefore skews biological interpretations derived from structural models (Carugo & Djinović Carugo, 2005 ▸; Pfanzagl *et al.*, 2020 ▸; Shelley & Garman, 2022 ▸; Bhattacharyya *et al.*, 2020 ▸). Consequently, detailed studies investigating radiation effects are essential to accurately interpret crystallographic structures and decipher the limitations imposed on structure interpretation by radiation damage (Gonzalez & Nave, 1994 ▸; Owen *et al.*, 2006 ▸; Kmetko *et al.*, 2006 ▸; Leal *et al.*, 2011 ▸; Sliz *et al.*, 2003 ▸; Borek *et al.*, 2007 ▸).

The core processes driving radiation damage are unavoidable. In addition to the elastic scattering of X-rays that gives rise to Bragg diffraction, inelastic scattering by photoelectric and Compton effects at any significant X-ray fluence causes damage (Nave, 1995 ▸). The susceptibility to radiation damage is linked to the composition of the crystalline samples and the solvent. For example, it has long been known that heavy-atom-containing crystals, those containing other phasing agents (such as selenomethionine) and heavy-metal buffers (such as arsenic in cacodylate buffer) actually increase radiation-damage sensitivity (Seltzer, 1993 ▸). The extent of radiation damage is both time- and temperature-dependent and is therefore partially mitigated by working at cryo-temperatures (∼100 K; Garman & Schneider, 1997 ▸). It is commonly accepted that at cryo-temperatures ‘global radiation damage’ can manifest itself as a progressive loss of high-resolution diffraction, an increase in *B* factors, unit-cell expansion and increased mosaicity (Holton, 2009 ▸). *Per se*, ‘global damage’ would not necessarily be visible directly in electron densities other than as a blurring effect. The notion of ‘specific damage’ includes reduction of metal centres, disulfide-bond cleavage, decarboxylation of aspartic and glutamic acid residues, and modifications of small-molecule ligands (for example dehalogenation; Ehler *et al.*, 2025 ▸), as well as small structural re­arrangements (for example side-chain movements) as a consequence of radiation-induced chemical modifications (Carugo & Djinović Carugo, 2005 ▸; Pfanzagl *et al.*, 2020 ▸; Gerstel *et al.*, 2015 ▸; Shelley & Garman, 2022 ▸; Bhattacharyya *et al.*, 2020 ▸; Garman & Weik, 2023 ▸).

Achieving sub-Ångström resolution in X-ray crystal structures is beneficial, since it allows structural models to be derived without any prior chemical knowledge nor stereochemical restraints during refinement for non-overlapping conformations, as a consequence of the high observable-to-refined parameter ratio in these resolution ranges (Blakeley *et al.*, 2015 ▸; Podjarny *et al.*, 2003 ▸; Dauter *et al.*, 1995 ▸). Benefits include the ability to visualize deviations from peptide planarity, distortions of the planarity of aromatic ring systems, subtle alternate-conformation networks, H-atom positions in protein and solvent, and a large proportion of the ordered solvent in fully and partially occupied positions. In addition, sub-Ångström resolution structures hold the promise of observing aspherical densities and other quantum-mechanical phenomena directly (Kleemiss *et al.*, 2021 ▸; Zarychta *et al.*, 2007 ▸; Guillot *et al.*, 2001 ▸; Woińska *et al.*, 2016 ▸). However, considering the adverse effects of radiation damage, the degree to which both global and specific radiation damage affect the details that can be discerned from biological structures, even at sub-Ångström resolution, currently remains unclear. To mitigate radiation damage affecting the interpretation of such structures, common practices include (i) the measurement of X-ray diffraction data at cryo-temperatures and (ii) restricting the ‘dose budget’ in data collection to preserve the measurability of weak high-angle reflections, taking into account the fading of the latter in response to dose (Borek *et al.*, 2007 ▸; Gonzalez & Nave, 1994 ▸; Owen *et al.*, 2006 ▸; Kmetko *et al.*, 2006 ▸; Ravelli & Garman, 2006 ▸; Leal *et al.*, 2011 ▸; Bourenkov & Popov, 2010 ▸; Fogh *et al.*, 2026 ▸). Automated data-collection strategies (Incardona *et al.*, 2009 ▸; Bourenkov & Popov, 2010 ▸) would typically suggest remaining below 1 MGy total dose for a sub-Ångström data set at 100 K. However, it has to be mentioned that these all represent empirical observations, with no absolute guidelines arising from systematic experiments. In fact, most systematic radiation-damage experiments are conducted at resolutions worse than 1.2 Å (Gonzalez & Nave, 1994 ▸; Owen *et al.*, 2006 ▸; Kmetko *et al.*, 2006 ▸; Leal *et al.*, 2011 ▸; Sliz *et al.*, 2003 ▸; Borek *et al.*, 2007 ▸), and to the best of our knowledge no systematic studies have been performed at sub-Ångström resolution. Therefore, there is an unmet need for systematic radiation-damage studies in this range of resolutions.

In this report, we address precisely this need and present the first radiation-damage study of the protein rubredoxin from *Pyrococcus abyssi* at resolutions ranging from 0.54 to 0.57 Å. We focus on the extent of information loss, model *B*-factor influence, the behaviour of a sensitive metal site, H atoms and how changes in electron density occur in the 0–1 MGy dose range that are relevant for macromolecular crystallography at sub-Ångström resolution.

## 2. Materials and methods

### 2.1. Rubredoxin purification, crystallization and crystal mounting

The synthetic open reading frame of rubredoxin (W4L, R5S) from *P. abyssi* was cloned into the T7-promoter-based vector pRSET A (Geneart, Regensburg, Germany). The six-histidine-tagged protein was produced in M9 medium in BL21-AI cells for 3 h at 37°C. The *Escherichia coli* pellet obtained by centrifugation of the culture at 5000*g* for 15 min was resuspended in 10 m*M* Tris–HCl pH 8.0, 10 m*M* imidazole, 200 m*M* NaCl, the cells were lysed by sonication and the lysate was cleared by centrifugation at 100 000*g*. Clear lysate was then loaded onto an Ni^2+^–NTA affinity column (Qiagen). After hydrolysis of the histidine tag by TEV protease, the protein was again loaded onto an Ni^2+^–NTA affinity column. In the last step, the protein was loaded onto a HiLoad 26/600 Superdex 75 pg column (Cytiva) equilibrated with 20 m*M* Tris–HCl pH 8.0, 150 m*M* NaCl, 0.1 m*M* EDTA. Finally, pure rubredoxin, as judged by SDS–PAGE and homogenous elution from gel-permeation chromatography, was concentrated to 36 mg ml^−1^ in 20 m*M* Tris–HCl pH 8.0 and stored at −80°C.

Crystals were grown in 3.6–3.8 *M* sodium malonate pH 6.0 using the sitting-drop vapour-diffusion method at 291 K. Crystals appeared after between one and two weeks. An orthorhombic crystal of *P. abyssi* rubredoxin with approximate dimensions of 320 × 240 × 200 µm was mounted in a LithoLoop (Molecular Dimensions) and directly cryo-cooled by plunging into liquid nitrogen.

### 2.2. X-ray data collection and processing

**Figure 1:**
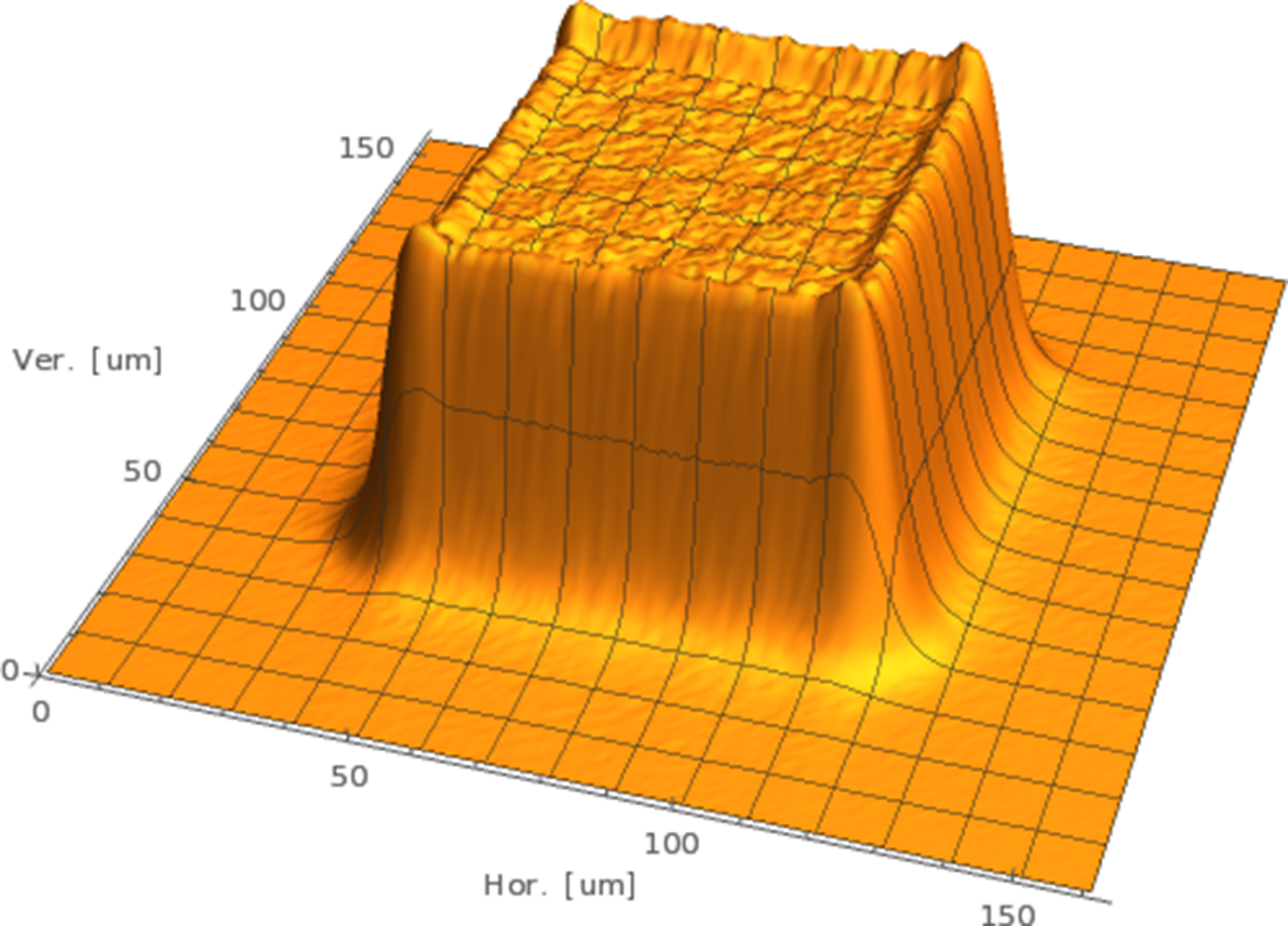
The typical beam profile of the top-hat beam at the P14 beamline is shown. The profile was measured using an Optique Peter (Lentilly, France) X-ray microscope. The effective pixel size is 0.65 µm. Slit diffraction features are visible at the edges of the beam. We therefore used a beam that was slightly larger than the crystal to account for the slit diffraction features.

Diffraction data were collected at 100 K at an energy of 26.718 keV (*i.e.* a wavelength of 0.4640 Å) with a 330 × 240 µm top-hat beam at full transmission on the EMBL Hamburg P14 beamline at the DESY PETRA III storage ring, Hamburg, Germany (Fig. 1 ▸). The top-hat beam, which was slightly larger than the crystal dimensions, was conditioned by a double-crystal monochromator and by 22 beryllium compound refractive lenses (500 µm apical radius), followed by proximal slits (20 cm from the sample) matching the largest crystal projection. Thus, homogeneous illumination of the crystal was achieved in all orientations during data collection, with a dose rate of 1.7 kGy s^−1^ as estimated by the program *RADDOSE*-3*D* version 5.0.1057 (Zeldin *et al.*, 2013 ▸; Bury *et al.*, 2018 ▸; Dickerson *et al.*, 2024 ▸). The beam profile was not measured in this particular experiment, but Fig. 1 ▸ shows a typical beam profile achieved in this setup, as measured using an X-ray imaging camera.

Diffraction data collection at these high energies was made possible by an EIGER2 CdTe 16M detector (Dectris, Baden, Switzerland; Donath *et al.*, 2023 ▸) that was placed at a distance of 139.5 mm from the sample position. Each dataset was collected in a total rotation range of 360° using an MD3down diffractometer (Arinax, Morains, France), with an image width of 0.1°. Two datasets were collected using a total dose [‘Average Dose (Whole Crystal)’ in *RADDOSE*-3*D* output, which in our case is equal to ‘Last Diffraction Weighted Dose’] of 50 kGy each, with an exposure corresponding to an absorbed dose of 900 kGy in between. We designate these two datasets as ‘50 kGy’ and ‘1 MGy’, respectively. Due to the opacity of the crystals, a colour change related to Fe^3+^ reduction was invisible within this dose range. Both datasets were collected with the same starting orientation.

Data were processed using *XDS* (Kabsch, 2010 ▸) within *autoPROC* (Vonrhein *et al.*, 2011 ▸), scaled using *AIMLESS* (Evans & Murshudov, 2013 ▸) and analysed further with *STARANISO* (Tickle *et al.*, 2018 ▸), without however applying anisotropic corrections to the merged reflection intensities. Diffraction limits were determined after applying a cutoff of 1.2 to the local average of *I*/σ(*I*), as implemented in *STARANISO*. The use of a crystal fully bathed in a top-hat beam guarantees that the accumulated dose is spatially uniform throughout the crystal, so that the contributions to the total intensity from all regions of the crystal are affected by the same decay *B* factor of typically 1 Å^2^ per MGy of accumulated dose. This dose-dependent decay is concordant with the *AIMLESS* scaling model involving restrained *B* factors for thin wedges of images, so that the correction of intensity decay through internal scaling can take place under optimal conditions.

### 2.3. Structure refinement

The unit-cell constants of the crystal were nearly identical to those of the previously determined structure of W4L, R5S *P. abyssi* rubredoxin (PDB entry 1yk4; Bönisch *et al.*, 2005 ▸). Therefore, we proceeded directly with refinement using this model in *BUSTER* (Bricogne *et al.*, 2017 ▸), using the *aB_autorefine* interface. Some alternate conformations were added in *Coot* (Emsley & Cowtan, 2004 ▸), and further refinement in *BUSTER*, using individual anisotropic atomic displacement parameters (ADPs) and occupancy refinement, yielded the final model, with the *R*_free_ flags being identical for the reflections common to the two datasets (Table 1 ▸).

In the final stage of maximum-likelihood refinement, positional parameters for all atoms were refined using typical restraints for bonds, angles, torsions, planes and nonbonded contacts. H atoms had an isotropic *B* factor refined (strongly restrained to the isotropic *B*-factor component of their parent atom), while all other atoms had anisotropic ADPs refined using several sphericity and similarity restraints (1–2, 1–3, 1–4 and nonbonded). The occupancy for atoms in alternate conformations (restrained to a sum to one), as well as those modelled as partially occupied (unrestrained in value), was also refined. The relative weights of the restraints acting upon these model parameters (*X*, *Y*, *Z*, *B*/ADP and occupancy) were left at the defaults used in *BUSTER* for any refinement. However, the weight on the X-ray term was adjusted to a relatively high value (29.0) typical for very high-resolution data. This automatic adjustment is based on the r.m.s.d. of observed bond distances relative to the standard EH99 values (Engh & Huber, 2006 ▸), with this value expected to be allowed to increase with higher resolution data, leading to a higher weight given to the X-ray data. Hydrogens were treated differently from other atoms in two respects according to the standard *BUSTER* ‘hybrid’ model for hydrogens: (i) the restraints active on them are significantly stronger and (ii) their bond distances are restrained to the nucleus position, except in the X-ray structure-factor calculation, where they are shifted to the electron cloud position.

Isomorphous difference Fourier maps 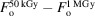 were calculated after scaling the amplitudes using *SCALEIT* from *CCP*4 (Howell & Smith, 1992 ▸; Agirre *et al.*, 2023 ▸) using the phases and weights from the ‘50 kGy’ refinement. The map coefficient MTZ file has been uploaded as supporting information and the maps from Fig. 3 can be reproduced from the column labelled 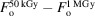 .

## 3. Results

The data statistics displayed in Table 1 ▸ show that both the ‘50 kGy’ and ‘1 MGy’ datasets are complete and have high signal-to-noise ratios. The unit-cell volume increased by 0.15% in the ‘1 MGy’ dataset and the mosaicity [*XDS* (Kabsch, 2010 ▸): e.s.d. reflection range] increased from 0.10° to 0.11° on average. Both datasets are slightly anisotropic, with the strongest diffraction along the *a* direction and weaker diffraction along the *b* and *c* axes. The level of anisotropy remains the same in both datasets. At an absorbed dose of 1 MGy, roughly 20% fewer reflections were observed than in the ‘50 kGy’ dataset. Models were refined with *BUSTER* to *R* factors of about 8% (Table 1 ▸). The average main-chain *B* factors were 3.6 Å^2^ for the ‘50 kGy’ dataset and 4.2 Å^2^ for the ‘1 MGy’ dataset. The *B* factors for the whole protein chain were found to be 4.9 Å^2^ in the ‘50 kGy’ dataset and increased to 5.8 Å^2^ in the ‘1 MGy’ dataset, which is in keeping with the rate of *B*-factor increase of 1 Å^2^ per MGy documented earlier (Borek *et al.*, 2007 ▸; Kmetko *et al.*, 2006 ▸; Leal *et al.*, 2011 ▸).

The four Fe–S distances of the FeS_4_ site of rubredoxin were found to be 2.282 (4) Å (Fe–Cys6 S^γ^), 2.257 (4) Å (Fe–Cys  S^γ^), 2.320 (4) Å (Fe–Cys39 S^γ^) and 2.262 (4) Å (Fe–Cys42 S^γ^) in the model refined against the ‘50 kGy’ dataset, an average of 2.28 Å. The standard uncertainty was estimated using the diffraction precision index (DPI) method based on *R*_free_ (Cruickshank, 1999 ▸; Blow, 2002 ▸; Gurusaran *et al.*, 2014 ▸); see Supplementary Table S1 for bond-error estimations based on other DPI calculation methods. These values agree well with EXAFS studies, which indicate an average value of 2.27 Å for the Fe^3+^–S distance (Bunker & Stern, 1977 ▸; Shulman *et al.*, 1978 ▸) for oxidized rubredoxin. In the model refined against the ‘1 MGy’ dataset, the four Fe–S distances were found to be marginally elongated to 2.324 (5) Å (Fe–Cys6 S^γ^), 2.291 (5) Å (Fe–Cys9 S^γ^), 2.353 (5) Å (Fe–Cys39 S^γ^) and 2.300 (5) Å (Fe-Cys42 S^γ^), an average of 2.32 Å. The standard uncertainty was estimated using the DPI method based on *R*_free_ (Cruickshank, 1999 ▸; Blow, 2002 ▸; Gurusaran *et al.*, 2014 ▸); see Supplementary Table S1 for bond-error estimations based on other DPI calculation methods. These values, on the other hand, would indicate that the iron is partially reduced to Fe^2+^ at ‘1 MGy’, in agreement with EXAFS data that indicate an average distance of 2.32 Å for Fe^2+^–S (Shulman *et al.*, 1978 ▸; Bunker & Stern, 1977 ▸). In Supplementary Table S1, we also calculate bond-length errors based on DPI for other rubredoxin structures in the Protein Data Bank (PDB; Berman *et al.*, 2000 ▸). This indicates that the Fe–S error estimates for the ‘50 kGy’ and ‘1 MGy’ structures reported here are by far the smallest.

**Figure 2:**
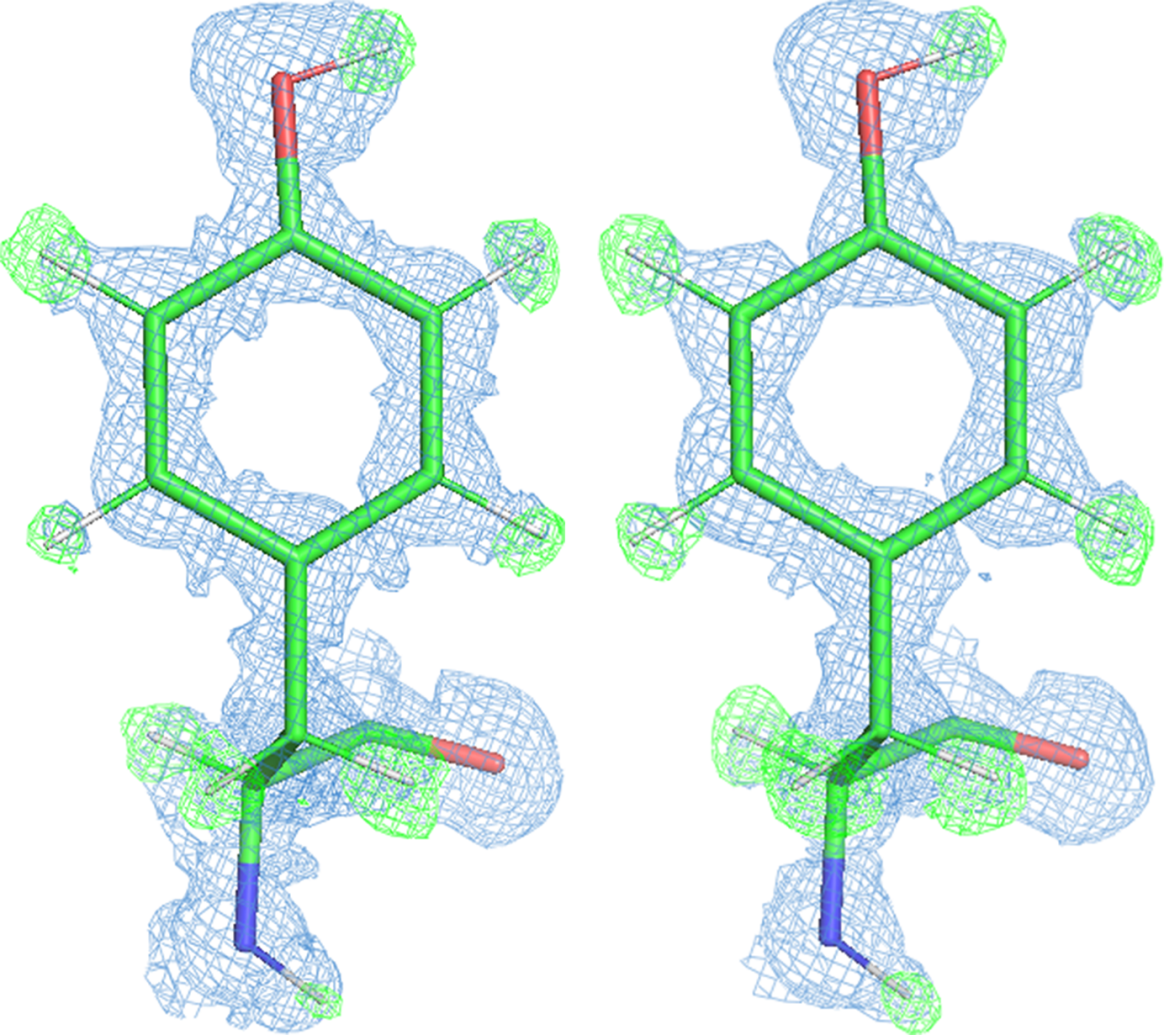
Tyr13 of rubredoxin is shown at 50 kGy (left) and 1 MGy (right) dose. The 2*mF*_o_ − *DF*_c_ densities (blue) are contoured at 0.5 e Å^−3^ and *mF*_o_ − *DF*_c_ hydrogen-omit densities (green) are contoured at 0.7 e Å^−3^. The 2*mF*_o_ − *DF*_c_ density centroids for hydrogens are closer to the parent atom, whereas *mF*_o_ − *DF*_c_ densities depict them closer to the nuclear position. H atoms are coloured grey and are placed at nuclear positions in the model.

*mF*_o_ − *DF*_c_ hydrogen-omit densities contoured at 0.7 e Å^−3^ display clearly visible hydrogen densities (Fig. 2 ▸). We noticed that the centroids of hydrogen positions in *mF*_o_ − *DF*_c_ hydrogen-omit maps of the 50 kGy structure appear closer to the nuclear distance of the H atoms (Fig. 2 ▸), in contrast to the librated position usually observed in high-resolution structures (Woińska *et al.*, 2016 ▸). 2*mF*_o_ − *DF*_c_ densities contoured at 0.5 e Å^−3^ of the ‘50 kGy’ structure are dominated by Fourier series-termination effects (Supplementary Fig. S1). The most prominent maxima arising from these effects appear at H-atom positions. Other local maxima at bond mid-points resemble bond electron density and valence bonds (Fig. 2 ▸, Supplementary Fig. S1). Such termination effects have been analysed in detail (Afonine *et al.*, 2004 ▸; Bochow & Urzhumtsev, 2005 ▸), and are expected to occur at resolutions of 0.4–0.6 Å and to affect atoms with isotropic *B*-factor equivalents of around 2 Å^2^. This is precisely the *B*-factor equivalent range of most protein atoms in the ‘50 kGy’ structure. These effects are known to mask aspherical density features (Afonine *et al.*, 2004 ▸; Bochow &Urzhumtsev, 2005 ▸). Notably, in the ‘1 MGy’ structure, they are much less pronounced, which agrees well with the increase in atomic displacement parameters (ADPs) by nearly 1 Å^2^. This supports the notion that ‘1 MGy’ might already represent too high a dose to investigate aspherical density features.

**Figure 3:**
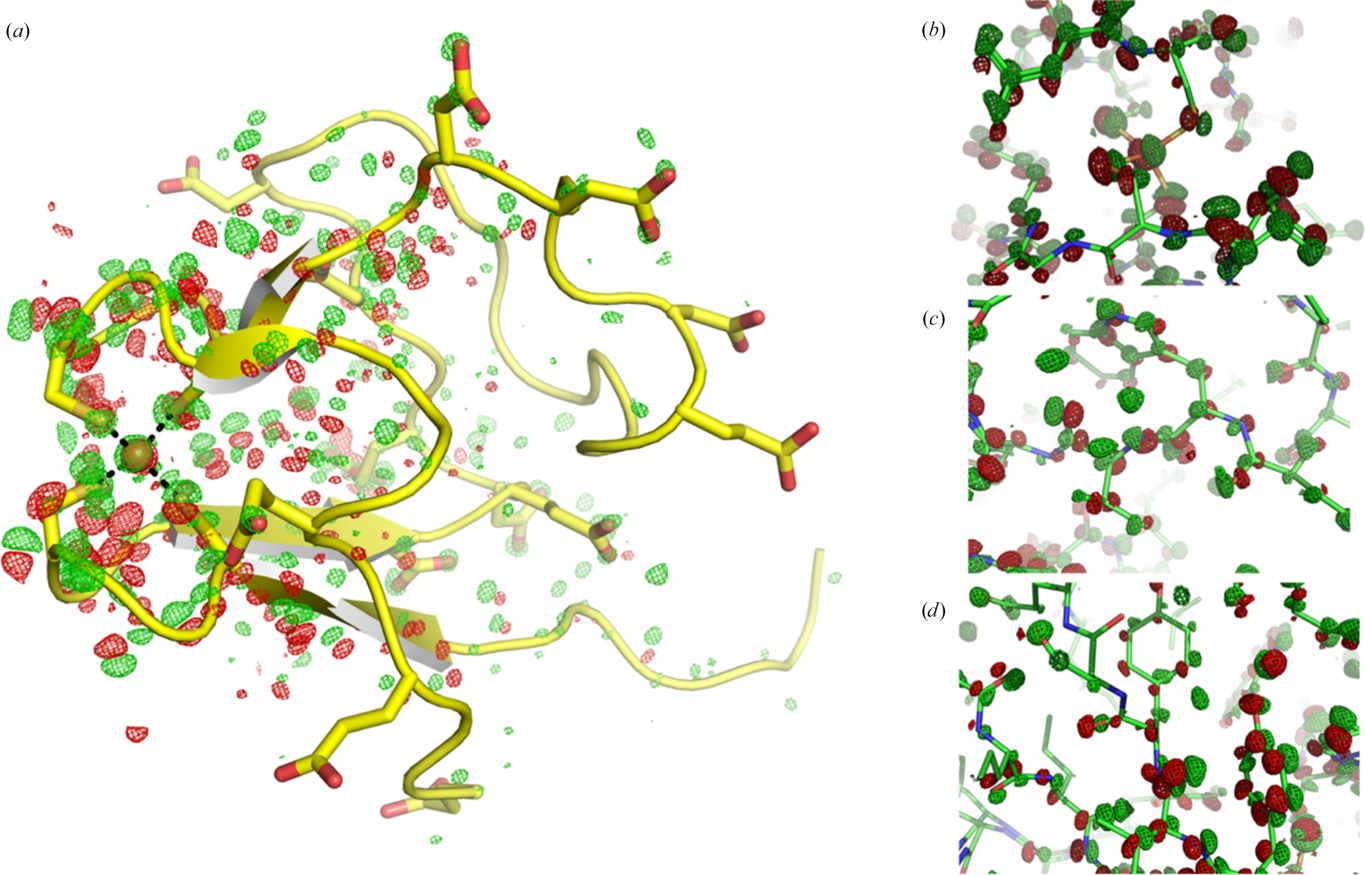
Isomorphous 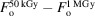 difference density is shown contoured at ±3.5 r.m.s. (positive density green, negative density red), revealing an extended conformational change that is most likely a consequence of iron reduction. (*a*) The backbone model of rubredoxin is shown; the side chains of cysteine, aspartate and glutamate residues are depicted as sticks. (*b*) Close-up view of the FeS_4_ site. (*c*) Close-up view of the vicinity of Trp37. (*d*) Close-up view of Tyr13.

Isomorphous difference Fourier maps of the ‘50 kGy’ and ‘1 MGy’ datasets reveal many interesting and unexpected features (Fig. 3 ▸). Fig. 3 ▸ shows 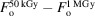 difference maps contoured at 3.5 r.m.s.. Inspection of these difference maps reveals that all positive and negative peaks at this contour level can be confidently interpreted in terms of small atomic displacements. These displacements are most prominent at the FeS_4_ site of rubredoxin and in the loops flanking these cysteine residues. They extend further into the adjacent β-sheets and decrease gradually towards the acidic periphery devoid of regular secondary structure. Therefore, the elongation of bonds at the rigid FeS_4_ site translates to long-range conformational changes, akin to hypothesized allosteric networks related to reactivity in enzymes. A more detailed view of the difference density (Figs. 3 ▸*b*–3 ▸*d*) shows that the atomic displacements are concerted within short residue stretches and can be described by small torsion-angle changes. Despite repeated attempts, we were not able to describe these atomic displacements in the form of an ensemble model because of difficulties in modelling the full connectivity of very small displacements. Instead, we used local modelling and occupancy refinement of several individual sites (not shown) to estimate the magnitudes of these displacements and the radiation-induced mobile fraction. This procedure allows us to estimate orders of magnitude of 0.1 Å for the displacements and of 10% for the mobile fraction.

**Figure 4:**
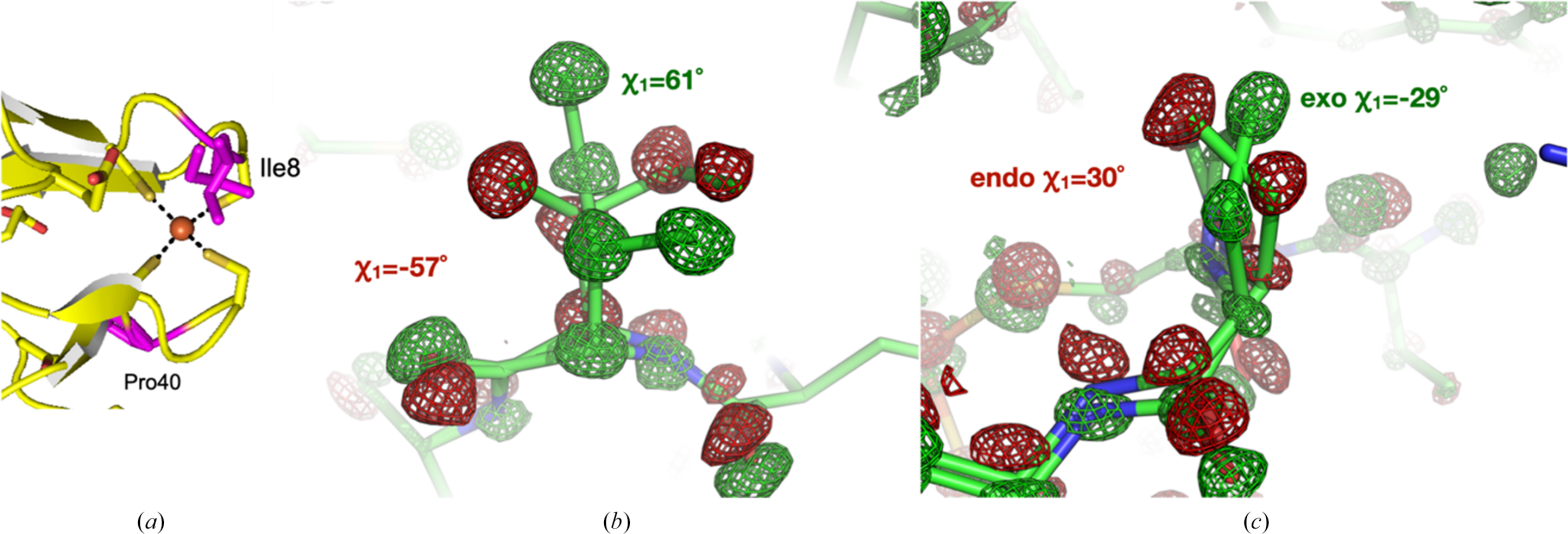
Isomorphous 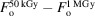 difference density is shown contoured at ±3.5 r.m.s. (positive density green, negative density red). (*a*) Positions of Ile8 and Pro40 (magenta) in relation to the FeS_4_ site. (*b*, *c*) Two alternate conformations of Ile8 and Pro40 interconvert in response to X-ray exposure.

Two important residues adjacent to the FeS_4_ site of rubredoxin, Ile8 and Pro40, adopt genuine alternate side-chain conformations (Fig. 4 ▸). In the ‘50 kGy’ structure, the occupancy of Ile8 conformer 1 (corresponding to the χ_1_ = −57° rotamer) is 45%, whereas that of conformer 2 (corresponding to the χ_1_ = 61° rotamer) is 55%. In the ‘1 MGy’ structure, conformers 1 and 2 are present with occupancies of 75% and 25%, respectively. Similarly, the *endo* χ_1_ = 30° and *exo* χ_1_ = −29° conformers of Pro40 interconvert with each other in a dose-dependent manner. While the occupancy in the ‘50 kGy’ structure is 40/60%, it is 55/45% in the ‘1 MGy’ structure. We speculate that an altered main-chain conformation may cause the rotamer exchange even at 100 K due to a low transition-energy barrier of the interconversion between rotameric states, as solvent rearrangements are not involved.

In summary, radiation-damage studies of rubredoxin at sub-Ångström resolution allow us to visualize discrete conformational changes in the 0–1 MGy dose regime that are likely to be correlated with the transition from oxidized to reduced rubredoxin.

## 4. Discussion

In this publication, we report what is to the best of our knowledge the first radiation-damage study on a protein at sub-Ångström resolution. We have utilized a collimated, top-hat beam on the EMBL P14 beamline of the DESY PETRA III storage ring to conduct our studies. The beam was slightly larger than the 300 × 240 × 200 µm crystal of *P. abyssi* rubredoxin. In our hands, the combination of large top-hat beams with high photon fluence at low dose rates in conjunction with large crystals is the only way to obtain reliable and high-quality sub-Ångström resolution data. This is demonstrated here by collecting one of the highest resolution (0.54 Å) X-ray diffraction datasets to date, using a dose of only 50 kGy. This capability of our experimental setup should serve as a reference for the future design of beamlines. Thus, contrary to our expectations, we succeeded in not only determining one of the highest resolution protein structures, but also one of the few (if not only) structures of rubredoxin where it is to a large extent in the oxidized state. This is apparent from the comparison between the Fe–S distance derived from both of our structures and the average EXAFS distances (Bunker & Stern, 1977 ▸; Shulman *et al.*, 1978 ▸). Comparison with rubredoxin structures from the PDB indicates that there is good agreement in Fe–S distances with PDB entry 1yk4, whereas other structures with comparable diffraction precision index (DPI) indicate elongated Fe—S bond distances (see also Supplementary Table S1).

We also collected a 50 kGy dataset on the same crystal after a 900 kGy ‘burn’, *i.e.* a ‘1 MGy’ dataset (950–1000 kGy dose), where we are able to confirm former findings of unit-cell expansions and mosaicity increase (Ravelli & McSweeney, 2000 ▸) that are insignificant in this dose range. However, 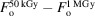 isomorphous difference Fourier maps reveal large regions where concerted directional atomic displacements occur in a dose-dependent manner. To our surprise, despite these atomic displacements, refinement of a rubredoxin model against the ‘1 MGy’ dataset yields a structure that is nearly indistinguishable in quality from the ‘50 kGy’ model. While the Fe–S distances are elongated, most likely indicating Fe^2+^—S bonds (Bunker & Stern, 1977 ▸; Clay *et al.*, 2002 ▸; Carugo & Djinović Carugo, 2005 ▸; Pfanzagl *et al.*, 2020 ▸; Shulman *et al.*, 1978 ▸), the anisotropic ADP refinement is able to nearly fully account for all the dose-dependent displacements in refinement. As described, the 2*mF*_o_ − *DF*_c_ electron-density maps are fraught with Fourier series-termination effects that diminish distinctly in the ‘1 MGy’ structure in comparison to the ‘50 kGy’ structure. This is in perfect agreement with the overall temperature-factor increase of 1 Å^2^ and associated blurring. We would like to point out that while diffraction data collected with a total accumulated dose of 1 MGy can be useful, it is likely that data collected with total doses above 0.5 MGy would not contribute significantly to accurate electron-density studies. In studies involving metalloproteins, it is clear that reduction of metal centres will occur, and a total dose of 100 kGy or less at 100 K is recommended (Carugo & Djinović Carugo, 2005 ▸; Pfanzagl *et al.*, 2020 ▸).


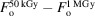
 isomorphous difference Fourier maps have revealed the occurrence of a dose-dependent extended conformational change. This change is particularly well resolved at the Ile8 and Pro40 sites, suggesting that radiation induces interconversion between two distinct states of rubredoxin. This involves almost the entire protein and is likely to be a consequence of reduction of the FeS_4_ site (Holton, 2009 ▸; Garman & Weik, 2023 ▸). With our sub-Ångström data, we are now able to observe discrete displacements of atomic positions (formerly described as ‘specific’ damage) that would otherwise be viewed as ‘global’ damage at lower resolution.

The key feature of the instrumental setup used here, namely a crystal fully bathed in a top-hat beam, is essential to ensure both a good decay correction by the *SCALA*/*AIMLESS* scaling model and a spatially uniform dose distribution throughout the crystal at any given time, so that the differences between the ‘50 kGy’ and ‘1 MGy’ structures are identical throughout the crystal and are not convoluted with the effects of spatial inhomogeneities in the dose distribution or the crystal illumination. We propose the notion and terminology of ‘resolution in dose’ to designate this extra benefit of our instrumental setup, whereby the crystal has at all times a well defined dose *value* rather than a broad dose *distribution*, and would thus describe this study as being ‘dose-resolved’.

We are aware that many of the observations described here may be specific to rubredoxin, but this illustrates the pressing need for systematic radiation-damage studies at sub-Ångström resolution involving a variety of systems diffracting to such resolution. The complete interpretation of extended dose-induced changes requires further studies (also into higher dose regimes) and the development of new computational tools to describe them.

## Supplementary Material

PDB reference: rubredoxin, 0.55 Å resolution, 50 kGy structure, 9ta4

PDB reference: 0.57 Å resolution, 1 MGy structure, 9ta6

Supplementary Figure and Table. DOI: 10.1107/S205979832600269X/xh5066sup1.pdf

Map coefficient MTZ file. DOI: 10.1107/S205979832600269X/xh5066sup2.zip

Diffraction images and autoPROC processing files.: https://10.5281/zenodo.18715183

## Figures and Tables

**Table 1:** Data-collection and refinement statistics Values in parentheses are for the highest resolution shell.

	‘50 kGy’ (0–50 kGy)	‘1 MGy’ (950–1000 kGy)
PDB code	9ta4	9ta6
Space group	*P*2_1_2_1_2_1_	*P*2_1_2_1_2_1_
*a*, *b*, *c* (Å)	24.793, 39.074, 44.895	24.808, 39.106, 44.898
α, β, γ (°)	90, 90, 90	90, 90, 90
Data collection		
Dose (kGy)	0–50	950–1000
Wavelength (keV/Å)	26.718/0.4640	26.718/0.4640
Resolution range (Å)	29.5–0.545 (0.586–0.545)	29.5–0.567 (0.628–0.567)
Anisotropic diffraction limits along *a*, *b* and *c* (Å)	0.544, 0.578, 0.690	0.579, 0.625, 0.650
Total No. of reflections	1596626	1311175
No. of unique reflections	117913	96302
Ellipsoidal completeness (%)	94.2 (59.5)	94.1 (57.9)
Spherical completeness (%)	80.9 (21.0)	74.1 (14.2)
Mean *I*/σ(*I*)	23.6 (1.7)	24.7 (1.7)
CC_1/2_	1.000 (0.705)	1.000 (0.713)
*R*_p.i.m._	0.014 (0.406)	0.013 (0.408)
Refinement		
*R*_work_/*R*_free_ (%)	7.63/8.51	7.57/8.41
No. of atoms		
Protein (including hydrogens)	1123	1123
Solvent	103	103
Average *B* factor (Å^2^)		
Protein (including hydrogens)	4.48	5.32
Solvent	15.5	17.4
Fe	1.97	2.41

## Data Availability

Structures were deposited in the PDB under accession codes 9ta4 (‘50 kGy’) and 9ta6 (‘1 MGy’). The diffraction images along with *autoPROC* processing files for ‘50 kGy’ and ‘1 MGy’ will be available for download from Zenodo at https://10.5281/zenodo.18715183.
