# Supplementary material for: Radiation damage in sub-Ångström resolution macromolecular crystallography: a low-dose study
Source: Acta Crystallogr D Struct Biol. 2026 Apr 13;82(Pt 5):484–91. doi: 10.1107/S205979832600269X (PMC13133993; doi:10.1107/S205979832600269X)
Supplement: Supplementary file 1 [file d-82-00484-sup1.pdf]

STRUCTURAL
BIOLOGY

Volume 82 (2026)

Supporting information for article:

Radiation damage in sub-ångström resolution macromolecular crystallography: a low-dose study

Gleb Bourenkov, Elham Paknia, Claus Flensburg, Rasmus Fogh, Peter Keller, Clemens Vonrhein, Gérard Bricogne and Ashwin Chari

Supplementary Figure S1: Fourier series termination effects at sub-Ångström resolution and low isotropic B-factor equivalents

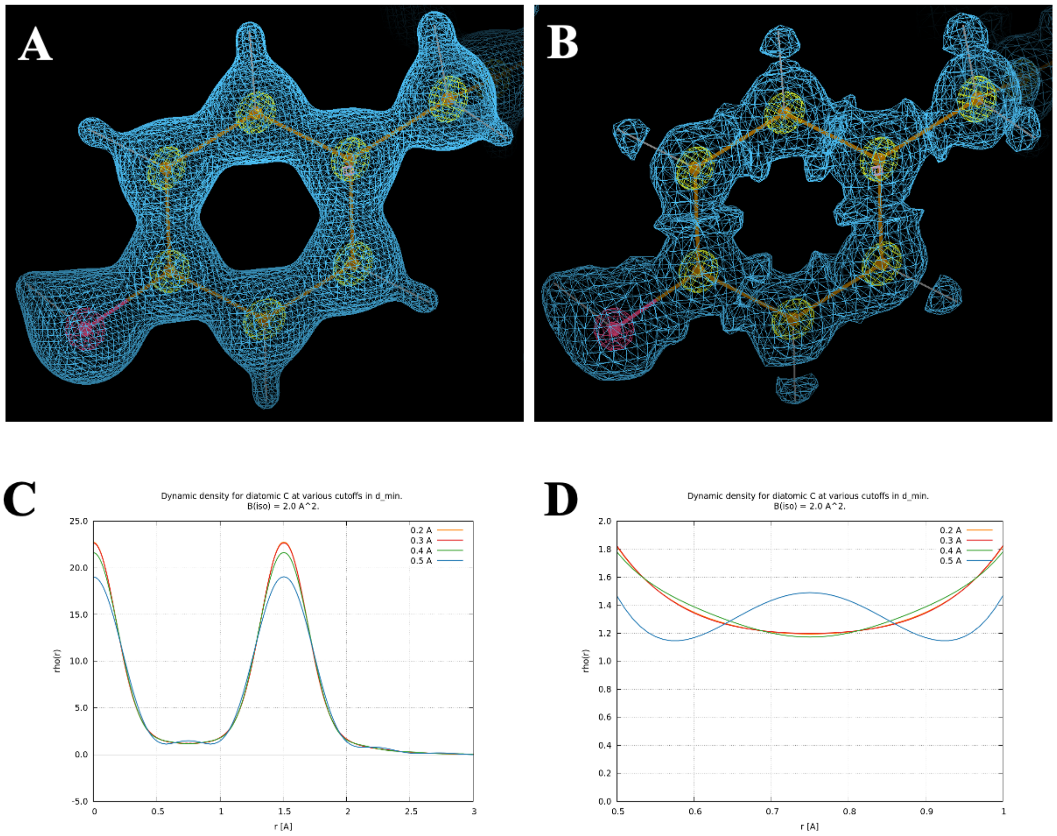

A) Shown is a F_{calc} map of Rubredoxin Tyr13 of the 50 kGy dataset using spherical independent atom model (IAM) scattering factors computed with a d_{min} = 0.3 Å and a grid of 300 x 480 x 540 points in the unit cell. The map is contoured at 0.7 e Å^{-3}. Fourier series termination effects are not visible.

B) The same as in A) computed with a dmin = 0.54 Å and a grid of 168 x 264 x 300 points in the unit cell. The map is contoured at 0.7 e Å^{-3}. Fourier series termination effects are clearly visible.

C) Calculated electron density along the line between two hypothetical carbon atoms with B_{iso} = 2.0 Å^2 placed 1.5 Å apart at different d_{min} cutoffs (yellow: 0.2 Å; red: 0.3 Å; green: 0.4 Å; blue: 0.5 Å).

D) The same as in C), but zoomed into the distance between 0.5 Å and 1 Å in between the carbon atoms. Note the local maximum arising at 0.75 Å distance in d_{min} = 0.5 Å as a consequence of Fourier series termination effects.

Supplementary Table S1: Bond distances of Fe-Cys-Sγ bonds in Rubredoxin structures:

9TA4 (50 kGy, this study)				
DPI positional error estimate	[1] DPI (Blow-10) based on R (Å)	[2] DPI (Blow-9) based on R_{free} (Å)	[3] DPI (Cruickshank) based on R (Å)	[4] DPI (Cruickshank) based on R_{free} (Å)
	0.00392	0.00430	0.00357	0.00389

Bond	Bond length (Å)	Bond length error based on [1] (Å)	Bond length error based on [2] (Å)	Bond length error based on [3] (Å)	Bond length error based on [4] (Å)
Fe-Cys6-Sγ	2.282	0.0037	0.0040	0.0033	0.0036
Fe-Cys9-Sγ	2.257	0.0036	0.0040	0.0033	0.0036
Fe-Cys39-Sγ	2.320	0.0035	0.0039	0.0032	0.0035
Fe-Cys42-Sγ	2.262	0.0036	0.0039	0.0033	0.0036

9TA6 (“1MGy”, this study)				
DPI positional error estimate	[1] DPI (Blow-10) based on R (Å)	[2] DPI (Blow-9) based on R_{free} (Å)	[3] DPI (Cruickshank) based on R (Å)	[4] DPI (Cruickshank) based on R_{free} (Å)
	0.00462	0.00504	0.00422	0.00456

Bond	Bond length (Å)	Bond length error based on [1] (Å)	Bond length error based on [2] (Å)	Bond length error based on [3] (Å)	Bond length error based on [4] (Å)
Fe-Cys6-Sγ	2.324	0.0044	0.0048	0.0040	0.0043
Fe-Cys9-Sγ	2.291	0.0044	0.0048	0.0040	0.0043
Fe-Cys39-Sγ	2.353	0.0042	0.0046	0.0038	0.0042
Fe-Cys42-Sγ	2.300	0.0043	0.0047	0.0039	0.0042

1BQ8

Bond	Bond length (Å)	Bond length error based on [1] (Å)	Bond length error based on [2] (Å)	Bond length error based on [3] (Å)	Bond length error based on [4] (Å)
Fe-Cys6-Sγ	2.285	0.0260	0.0294	0.0212	0.0248
Fe-Cys9-Sγ	2.260	0.0267	0.0302	0.0218	0.0254
Fe-Cys39-Sγ	2.306	0.0260	0.0294	0.0212	0.0248
Fe-Cys42-Sγ	2.266	0.0270	0.0305	0.0220	0.0257

1BQ9

Bond	Bond length (Å)	Bond length error based on [1] (Å)	Bond length error based on [2] (Å)	Bond length error based on [3] (Å)	Bond length error based on [4] (Å)
Fe-Cys6-Sγ	2.274	0.0477	0.0511	0.0400	0.0444
Fe-Cys9-Sγ	2.256	0.0486	0.0521	0.0408	0.0453
Fe-Cys39-Sγ	2.292	0.0476	0.0510	0.0399	0.0444
Fe-Cys42-Sγ	2.256	0.0486	0.0521	0.0407	0.0453

1BRF					
Bond	Bond length (Å)	Bond length error based on [1] (Å)	Bond length error based on [2] (Å)	Bond length error based on [3] (Å)	Bond length error based on [4] (Å)
Fe-Cys6-Sγ	2.299	0.0227	0.0263	0.0188	0.0222
Fe-Cys9-Sγ	2.265	0.0230	0.0266	0.0191	0.0225
Fe-Cys39-Sγ	2.314	0.0224	0.0260	0.0186	0.0219
Fe-Cys42-Sγ	2.280	0.0229	0.0265	0.0190	0.0224
1IU5					
Bond	Bond length (Å)	Bond length error based on [1] (Å)	Bond length error based on [2] (Å)	Bond length error based on [3] (Å)	Bond length error based on [4] (Å)
Fe-Cys6-Sγ	2.268	0.0754	0.0713	0.0704	0.0679
Fe-Cys9-Sγ	2.263	0.0795	0.0752	0.0743	0.0716
Fe-Cys39-Sγ	2.305	0.0763	0.0723	0.0714	0.0688
Fe-Cys42-Sγ	2.262	0.0781	0.0740	0.0731	0.0705
1YK4					
Bond	Bond length (Å)	Bond length error based on [1] (Å)	Bond length error based on [2] (Å)	Bond length error based on [3] (Å)	Bond length error based on [4] (Å)
Fe-Cys6-Sγ	2.295	0.0057	0.0061	0.0054	0.0057
Fe-Cys9-Sγ	2.273	0.0058	0.0061	0.0055	0.0057
Fe-Cys39-Sγ	2.333	0.0055	0.0058	0.0052	0.0055
Fe-Cys42-Sγ	2.277	0.0056	0.0059	0.0053	0.0056
1YK5					
Bond	Bond length (Å)	Bond length error based on [1] (Å)	Bond length error based on [2] (Å)	Bond length error based on [3] (Å)	Bond length error based on [4] (Å)
Fe-Cys6-Sγ (chain A)	2.298	0.1122	0.1111	0.1053	0.1067
Fe-Cys9-Sγ (chain A)	2.222	0.1128	0.1116	0.1058	0.1073
Fe-Cys39-Sγ (chain A)	2.305	0.1141	0.1129	0.1071	0.1086
Fe-Cys42-Sγ (chain A)	2.210	0.1166	0.1153	0.1093	0.1109
Fe-Cys6-Sγ (chain B)	2.310	0.1060	0.1049	0.0994	0.1008
Fe-Cys9-Sγ (chain B)	2.276	0.1065	0.1053	0.0999	0.1012
Fe-Cys39-Sγ (chain B)	2.285	0.1055	0.1044	0.0990	0.1003
Fe-Cys42-Sγ (chain B)	2.202	0.1090	0.1078	0.1022	0.1036

1YK5 (continued)					
Fe-Cys6-Sγ (chain C)	2.243	0.1056	0.1045	0.0991	0.1005
Fe-Cys9-Sγ (chain C)	2.233	0.1082	0.1070	0.1015	0.1029
Fe-Cys39-Sγ (chain C)	2.271	0.1091	0.1080	0.1023	0.1038
Fe-Cys42-Sγ (chain C)	2.286	0.1116	0.1105	0.1047	0.1062
Fe-Cys6-Sγ (chain D)	2.282	0.1125	0.1113	0.1055	0.1070
Fe-Cys9-Sγ (chain D)	2.230	0.1129	0.1117	0.1059	0.1074
Fe-Cys39-Sγ (chain D)	2.258	0.1161	0.1149	0.1089	0.1104
Fe-Cys42-Sγ (chain D)	2.277	0.1188	0.1175	0.1114	0.1130
2PYA					
Bond	Bond length (Å)	Bond length error based on [1] (Å)	Bond length error based on [2] (Å)	Bond length error based on [3] (Å)	Bond length error based on [4] (Å)
Fe-Cys6-Sγ	2.309	0.0094	0.0097	0.0089	0.0090
Fe-Cys9-Sγ	2.290	0.0093	0.0096	0.0088	0.0089
Fe-Cys39-Sγ	2.345	0.0090	0.0092	0.0084	0.0086
Fe-Cys42-Sγ	2.296	0.0092	0.0095	0.0087	0.0088
3KYU					
Bond	Bond length (Å)	Bond length error based on [1] (Å)	Bond length error based on [2] (Å)	Bond length error based on [3] (Å)	Bond length error based on [4] (Å)
Fe-Cys5-Sγ	2.313	0.0243	0.0262	0.0220	0.0231
Fe-Cys8-Sγ	2.273	0.0252	0.0272	0.0228	0.0240
Fe-Cys38-Sγ	2.323	0.0238	0.0257	0.0216	0.0227
Fe-Cys41-Sγ	2.297	0.0245	0.0265	0.0223	0.0234
3KYV					
Bond	Bond length (Å)	Bond length error based on [1] (Å)	Bond length error based on [2] (Å)	Bond length error based on [3] (Å)	Bond length error based on [4] (Å)
Fe-Cys5-Sγ	2.324	0.0272	0.0290	0.0250	0.0260
Fe-Cys8-Sγ	2.282	0.0279	0.0298	0.0257	0.0268
Fe-Cys38-Sγ	2.332	0.0268	0.0286	0.0247	0.0257
Fe-Cys41-Sγ	2.313	0.0276	0.0295	0.0254	0.0265

3KYW					
Bond	Bond length (Å)	Bond length error based on [1] (Å)	Bond length error based on [2] (Å)	Bond length error based on [3] (Å)	Bond length error based on [4] (Å)
Fe-Cys5-Sγ	2.292	0.0297	0.0305	0.0287	0.0285
Fe-Cys8-Sγ	2.270	0.0309	0.0317	0.0298	0.0296
Fe-Cys38-Sγ	2.304	0.0296	0.0304	0.0286	0.0284
Fe-Cys41-Sγ	2.262	0.0304	0.0312	0.0294	0.0292
3KYX					
Bond	Bond length (Å)	Bond length error based on [1] (Å)	Bond length error based on [2] (Å)	Bond length error based on [3] (Å)	Bond length error based on [4] (Å)
Fe-Cys5-Sγ	2.299	0.1162	0.1121	0.1704	0.1174
Fe-Cys8-Sγ	2.307	0.1201	0.1159	0.1761	0.1213
Fe-Cys38-Sγ	2.296	0.1151	0.1110	0.1687	0.1162
Fe-Cys41-Sγ	2.306	0.1197	0.1155	0.1755	0.1209
3KYY					
Bond	Bond length (Å)	Bond length error based on [1] (Å)	Bond length error based on [2] (Å)	Bond length error based on [3] (Å)	Bond length error based on [4] (Å)
Fe-Cys5-Sγ	2.298	0.0234	0.0312	0.1002	0.0681
Fe-Cys8-Sγ	2.272	0.0243	0.0324	0.1040	0.0707
Fe-Cys38-Sγ	2.308	0.0233	0.0310	0.0996	0.0677
Fe-Cys41-Sγ	2.263	0.0240	0.0319	0.1026	0.0697
4AR6					
Bond	Bond length (Å)	Bond length error based on [1] (Å)	Bond length error based on [2] (Å)	Bond length error based on [3] (Å)	Bond length error based on [4] (Å)
Fe-Cys5-Sγ	2.338	0.0151	0.0154	0.0155	0.0154
Fe-Cys8-Sγ	2.308	0.0156	0.0159	0.0159	0.0158
Fe-Cys38-Sγ	2.346	0.0151	0.0154	0.0154	0.0153
Fe-Cys41-Sγ	2.313	0.0155	0.0158	0.0158	0.0157
5NW3					
Bond	Bond length (Å)	Bond length error based on [1] (Å)	Bond length error based on [2] (Å)	Bond length error based on [3] (Å)	Bond length error based on [4] (Å)
Fe-Cys5-Sγ	2.302	0.0040	0.0043	0.0040	0.0042
Fe-Cys8-Sγ	2.270	0.0041	0.0044	0.0041	0.0043
Fe-Cys38-Sγ	2.319	0.0040	0.0042	0.0039	0.0042
Fe-Cys41-Sγ	2.284	0.0040	0.0043	0.0040	0.0043

5OME					
Bond	Bond length (Å)	Bond length error based on [1] (Å)	Bond length error based on [2] (Å)	Bond length error based on [3] (Å)	Bond length error based on [4] (Å)
Fe-Cys5-Sγ	2.345	0.0067	0.0071	0.0066	0.0069
Fe-Cys8-Sγ	2.304	0.0069	0.0074	0.0069	0.0072
Fe-Cys38-Sγ	2.352	0.0066	0.0070	0.0066	0.0069
Fe-Cys41-Sγ	2.322	0.0068	0.0072	0.0068	0.0071
9BKP					
Bond	Bond length (Å)	Bond length error based on [1] (Å)	Bond length error based on [2] (Å)	Bond length error based on [3] (Å)	Bond length error based on [4] (Å)
Fe-Cys6-Sγ	2.286	0.1351	0.1196	0.1284	0.1170
Fe-Cys9-Sγ	2.245	0.1367	0.1210	0.1300	0.1184
Fe-Cys39-Sγ	2.333	0.1300	0.1151	0.1236	0.1126
Fe-Cys42-Sγ	2.270	0.1446	0.1280	0.1375	0.1253
9BKT					
Bond	Bond length (Å)	Bond length error based on [1] (Å)	Bond length error based on [2] (Å)	Bond length error based on [3] (Å)	Bond length error based on [4] (Å)
Fe-Cys6-Sγ	2.361	0.1202	0.1053	0.0717	0.0763
Fe-Cys9-Sγ	2.374	0.1216	0.1065	0.0725	0.0772
Fe-Cys39-Sγ	2.409	0.1131	0.0991	0.0674	0.0718
Fe-Cys42-Sγ	2.366	0.1191	0.1044	0.0710	0.0756

[1] DPI(Blow-10) based on R
[2] DPI(Blow-9) based on Rfree
[3] DPI(Cruickshank) based on R
[4] DPI(Cruickshank) based on Rfree

References:

Blow, D. M. (2002). *Acta Crystallogr. D Biol. Crystallogr.* **D58**, 792–797.

Cruickshank, D. W. J. (1999). *Acta Crystallogr. D Biol. Crystallogr.* **55**, 583–601.

Gurusaran, M., Shankar, M., Nagarajan, R., Helliwell, J. R. & Sekar, K. (2014). *IUCrJ* **1**, 74–81.
